# HDAC Inhibition Elicits Myocardial Protective Effect through Modulation of MKK3/Akt-1

**DOI:** 10.1371/journal.pone.0065474

**Published:** 2013-06-10

**Authors:** Ting C. Zhao, Jianfeng Du, Shugang Zhuang, Paul Liu, Ling X. Zhang

**Affiliations:** 1 Cardiovascular Research laboratory, Department of Surgery, Roger Williams Medical Center, Boston University Medical School, Providence, Rhode Island, United States of America; 2 Department of Medicine, Rhode Island Hospital, Brown Medical School, Brown University, Providence, Rhode Island, United States of America; 3 Department of Plastic Surgery, Rhode Island Hospital, Brown Medical School, Brown University, Providence, Rhode Island, United States of America; Virginia Commonwealth University Medical Center, United States of America

## Abstract

We and others have demonstrated that HDAC inhibition protects the heart against myocardial injury. It is known that Akt-1 and MAP kinase play an essential role in modulation of myocardial protection and cardiac preconditioning. Our recent observations have shown that Akt-1 was activated in post-myocardial infarction following HDAC inhibition. However, it remains unknown whether MKK3 and Akt-1 are involved in HDAC inhibition-induced myocardial protection in acute myocardial ischemia and reperfusion injury. We sought to investigate whether the genetic disruption of Akt-1 and MKK3 eliminate cardioprotection elicited by HDAC inhibition and whether Akt-1 is associated with MKK3 to ultimately achieve protective effects. Adult wild type and MKK3^−/−^, Akt-1^−/−^ mice received intraperitoneal injections of trichostatin A (0.1mg/kg), a potent inhibitor of HDACs. The hearts were subjected to 30 min myocardial ischemia/30 min reperfusion in the Langendorff perfused heart after twenty four hours to elicit pharmacologic preconditioning. Left ventricular function was measured, and infarct size was determined. Acetylation and phosphorylation of MKK3 were detected and disruption of Akt-1 abolished both acetylation and phosphorylation of MKK3. HDAC inhibition produces an improvement in left ventricular functional recovery, but these effects were abrogated by disruption of either Akt-1 or MKK3. Disruption of Akt-1 or MKK3 abolished the effects of HDAC inhibition-induced reduction of infarct size. Trichostatin A treatment resulted in an increase in MKK3 phosphorylation or acetylation in myocardium. Taken together, these results indicate that stimulation of the MKK3 and Akt-1 pathway is a novel approach to HDAC inhibition -induced cardioprotection.

## Introduction

Histone acetyltransferases (HAT) and histone deacetylases (HDAC) have recently garnered attention because they have emerged as important mechanisms in the regulation of a variety of cellular responses. Histone acetylation is mediated by histone acetyltransferase, which results in the modification of the structure of chromatin leading to nucleosomal relaxation and altered transcriptional activation. In contrast, the reverse reaction is mediated by histone deacetylase which induces deacetylation, chromatin condensation, and transcriptional repression [1]–[5]. The acetylation status of histone tails is determined by the interplay between HATs and HDACs. The opposing actions of HAT and HDACs allow for gene expression to be exquisitely regulated through chromatin remodeling.

Since the identification of HDAC 1 (named HD 1) [6], over a dozen HDACs have been described in mammals [7]. These HDACs can be categorized into three distinct classes. Class I HDACs consist of HDAC 1, 2, 3, and 8, which are ubiquitously expressed and predominantly located in nuclei. Class II HDACs include HDAC 4, 5, 7, and 9. In contrast to class I, class II HDACs exhibit a tissue specific pattern of expression. HDAC 4 and HDAC 5 are highly expressed in the heart, brain, and skeletal muscle and shuttle between the nucleus and cytoplasm [8]–[11]. We have recently demonstrated that the inhibition of histone deacetylase with a selective inhibitor, trichostatin A, showed cardioprotective effects against I/R injury[12]. This is consistent with the observations showing that the inhibition of HDAC in myocytes silences fetal gene activation, blocks cardiac hypertrophy, and prevents cardiac remodeling [13]–[15]. Furthermore, HDAC inhibition has previously been shown to markedly decrease infarct size and reduce ischemia-induced neurological deficit scores in focal cerebral ischemia model of rats [16]. Our recently observations suggest that HDAC inhibition protected the heart against myocardial ischemia injury and prevented remodeling.

The Akt family of intracellular protein kinases regulates cellular growth, proliferation, and metabolism in many systems. Cardiac development and postnatal growth depend on the activation of Akt. Observations from our laboratories demonstrate elevated levels of phosphatidylinositol 3-kinase (PI3K) and Akt kinases during the proliferative period of cardiac growth [17]. It is well known that Akt serves as a powerful survival signal to protect the heart against myocardial injury [18]–[20]. The activation of Akt signaling in bone marrow derived mesenchymal stem cells resulted in the prevention of cardiac remodeling, an increase in regenerated myocardium, and angiogenesis and restoration of myocardial function [21]–[24]. In our previous studies, we utilized a unique and established stem cell engineered approach to deliver *Wt* and Akt-1^−/−^ lin^-^c-kit^+^ stem cells following myocardial infarction [25]. Our results demonstrate that peripheral administration of armed lin^-^c-kit^+^ cells restores myocardial function and promotes an angiogenic response, which is dependent upon Akt-1 signaling pathway. In addition, it is well established that stimulation of MAP kinases is closely associated with the prevention of myocardial ischemia and reperfusion injury. p38, as a downstream target of MKK3, has been demonstrated to be essential in development of protective effects following stimulation of unique signaling pathway [26], [27]. However, it remains unknown whether HDAC inhibition modulates myocardial ischemia and reperfusion injury through MKK3 and/or Akt-1. In these studies, we investigated: 1) whether the cardioprotective effects induced with HDAC inhibitor, TSA, could be diminished with the targeted deletion of either MKK3; 2) whether the cardioprotective effects induced with HDAC inhibitor, TSA, could be diminished with the targeted deletion of Akt-1; 3) whether HDAC inhibition would enhance MKK3 acetylation as well as phosphorylation in HDAC inhibition-preconditioned myocardium, and whether MKK3 phosphorylation and acetylation required the existence of Akt-1. To the best of our knowledge, this is the first study to provide new insight into our understanding of HDAC inhibition-induced cardioprotective effect that involves MKK3 and Akt-1 and thereby reveals novel mechanisms of ischemic injury to develop therapeutic strategies for heart disease.

## Materials and Methods

### Animals

Adult male B6.129 wild type and MKK3^−/−^and Akt-1^−/−^ mice were obtained from Jackson Laboratory (Bar Harbor, Maine). All animal experiments were conducted under a protocol approved by the Institutional Animal Care and Use Committee of Rhode Island Hospital, which conforms to the Guide for the Care and Use of Laboratory Animals published by the US National Institutes of Health (NIH Publication No. 85–23, revised 1996).

### Langendorff Isolated Heart Perfusion

The methodology of Langendorff**’**s isolated perfused heart preparation has been described previously in detail [28]–[31]. Briefly, mice were anesthetized with an intraperitoneal injection (i.p.) of pentobarbital sodium (120 mg/kg). The hearts were rapidly excised and arrested in ice-cold Krebs-Henseleit buffer. They were then cannulated via the ascending aorta for retrograde perfusion by the Langendorff method using Krebs-Henseleit buffer containing (in mM) 110 NaCl, 4.7 KCl, 1.2 MgSO_4_ 7H_2_O, 2.5 CaCl_2_ 2H_2_O, 11 glucose, 1.2 KH_2_PO_4_, 25 NaHCO_3_, and 0.5 EDTA. The buffer, aerated with 95% O_2_∶5% CO_2_ to give a pH of 7.4 at 37°C, was perfused at a constant pressure of 55 mmHg. A water-filled latex balloon, attached to the tip of polyethylene tubing, was then inflated sufficiently to provide a left ventricular end-diastolic pressure (LVEDP) of about 10 mmHg. Myocardial function was measured including left ventricular developed pressure (LVDP), LVEDP, RPP, heart rate, and coronary flow. LVDP was calculated by subtracting LVEDP from peak systolic pressure. Rate pressure product (RPP), an index of cardiac work, was calculated by multiplying LVDP with heart rate.

### Measurement of Myocardial Infarction

The infarction size was measured with a modification as previously described [29], [30]. At the end of reperfusion, hearts were perfused with 10% triphenyltetrazolium chloride (TTC), and then removed from the Langendorff perfusion apparatus. The frozen hearts were then cut from apex to base into transverse slices. After staining, 10% TTC buffer was replaced, and then the slices were fixed in formaldehyde for measurement of the infarcted areas using computer morphometry NIH image software (Image *J* 1. 36, NIH). The infarct size was calculated and presented as the percentage of risk area, defined as the sum of total ventricular area minus cavities.

### Experimental Protocol

**Figure 1:**
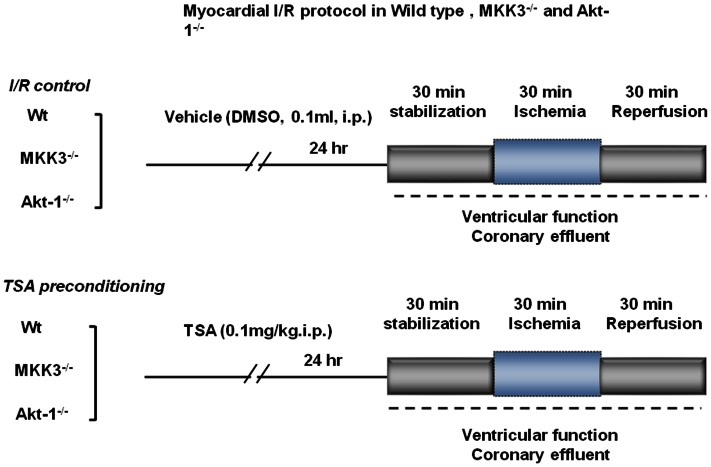
The experimental protocol. Wt: wild-type, TSA: trichostatin A; I/R: ischemia/reperfusion; DMSO: dimethyl sulfoxide.

Mice were randomized into six experimental groups that underwent the following treatments, as shown in **Figure 1**∶1) Vehicle group: wild-type mice receiving an i.p. injection of 0.1 ml vehicle (Dimethyl sulfoxide, 5% DMSO); 2) TSA+wild-type group: wild-type animals were treated the same as group 1 except that trichostatin A (TSA, 0.1mg/kg, i.p.) was given to wild-type animals; 3) TSA+ Akt-1^−/−^ group: the same as group 2 except that Akt-1^−/−^ mice were administered with TSA; 4) Akt-1^−/−^ group: the same as group 3 except that Akt-1^−/−^ mice were administered with vehicle instead of TSA; 5) MKK3^−/−^+TSA: the same as group 2 except that MKK3^−/−^ mice were administered with TSA; 6) MKK3^−/−^ mice group: the same as group 5 except that MKK3^−/−^ mice were administered with vehicle instead of TSA. Twenty-four hours later, the hearts were subjected to 30 min of stabilization and 30 min of ischemia followed by 30 min of reperfusion.

Another subset of animals including wild type and Akt-1^−/−^ backgrounds without sustained ischemia and reperfusion were treated with or without TSA solely for the purpose of measuring MKK3 acetylation and phosphorylation. Animals were treated with TSA for 30 min, and heart tissues were collected. Cardiac lysates were extracted. Briefly, the hearts were frozen in liquid nitrogen, ground, and suspended in 1 ml of lysis buffer containing 50 mM Tris HCl (pH 7.4), 0.1 mM sodium orthovanadate, 50 mM sodium fluoride, 150 mM sucrose, 1 mM phenylmethylsulfonyl fluoride (PMSF), 5 mM EDTA, 5 mM EGTA, 2 µg/ml leupeptin, 2 µg/ml aprotinin, and 5 µg/ml pepstatin A. Mixtures were homogenized and microcentrifuged at 14,000 rpm for 20 min. The protein content of the supernatant was determined using the detergent compatible-protein assay (Bio-Rad).

### Immunoprecipitation and Western Blot Analysis

Protein G immunoprecipitation protocol was performed according to the manufacturer’s instruction from Sigma. Protein lysates (100µg) were incubated with 1 µg of anti-MKK3 antibody and protein G agarose beads (30 µl per each reaction) to bind the antibody. The proteins from wild type and Akt-1^−/−^ mice were detected using MKK3, phosphorylated polycolonal anti-MKK3, and anti-lysine antibodies from Cell Signaling™ (Danvers, MA). For immunoblotting, proteins (50µg/lane) were separated by 10% SDS-PAGE. Proteins were then transferred onto a nitrocellulose membrane for 2 h at 100 Volts. The membrane was blocked with 5% non-fat dry milk in 1X Tris-buffered saline containing 0.5% Tween 20 for 1 h. The blots were incubated with respective primary antibodies (1∶1,000 dilution) for 2 h and visualized by incubation with anti-rabbit horseradish peroxidase-conjugated secondary antibody (1∶5,000 dilution) for 1 h. The immunoblots were developed with the ECL Chemiluminescence Detection Reagent (Amersham Pharmacia Biotech). The densitometric results were normalized to the control group and expressed as percentages of control values.

### Immunofluorescent Staining

Cardiac tissues were snap-frozen in pre-chilled 2-methylbutane and embedded in TBS (Pittsburgh, PA). Ten µm-thick sections were cut with a cryostat (Thermo Shandon, Pittsburgh, USA) at −22°C, mounted on plus charge slides (Fisher Scientific) and air-dried at room temperature. Sections were fixed in 3.7% (vol/vol) paraformaldehyde for 15 min and permeabilized in 0.5% Triton X-100 in PBS for 10 min. The sections were then incubated with anti-α-sarcomeric actininmonoclonal antibody (vWF, Sigma, St. Louis, MO) and anti-histone deacetylase 4 and 5 antibody for 1 hr. Secondary antibodies anti-mouse IgM Fluorescein and anti-rabbit IgG-Cy3 (Vector Laboratories, Burlingame, CA) were applied at room temperature. Fluorescent imaging was performed using a high-resolution *ZEISS LSM-700* fluorescence microscope equipped with ZEN2009 software for image analysis.

### Statistics

All measurements are expressed as means ± SE. Difference among the groups were analyzed by two-way analysis of variance (ANOVA), followed by post hoc Bonferroni correction or Student’s unpaired *t* test for two groups. The analysis examines the effects of two variables (pharmacological treatments: vehicle and TSA; mouse genetic backgrounds: wild type, MKK3^−/−^ and Akt-1^−/−^). Statistical differences were considered significant with a value of p<0.05.

## Results

### Pre-ischemic Ventricular Functional Parameters

Baseline cardiac functions including LVSP, LVEDP, LVDP, rate pressure product, and heart rate were recorded among the groups. As shown in Table 1, there were no differences among the groups before ischemia.

**Table 1:** Baseline ventricular function.

Groups	LVSP (mmHg)	LVEDP (mmHg)	RPP (mmHg/min×10^3^)	CF (ml/min)	HR (beats/min)
WT	118±30	12±4	35±8	3.5±0.2	333±7
TSA+WT	120±26	12±7	33±7	4.2±0.4	339±49
MKK3^−/−^	112±10	10±7	35±3	4.1±0.3	349±18
TSA+MKK3^−/−^	130±14	13±5	38±2	3.1±0.1	330±14
Akt-1^−/−^	112±11	3±3	27±2	3.3±0.3	377±36
TSA+Akt-1^−/−^	102±10	4±2	41±4	3.9±0.4	424±

LVSP: Left ventricular systolic pressure; LVEDP: Left ventricular end-diastolic pressure; RPP: Rate pressure product; CF: coronary effluent; HR: heart rate; TSA: trichostatin A. No significant differences were found between the experimental groups for any of the functional parameters (n = 4–5/per group).

### Post-ischemic Myocardial Infarct Size

**Figure 2:**
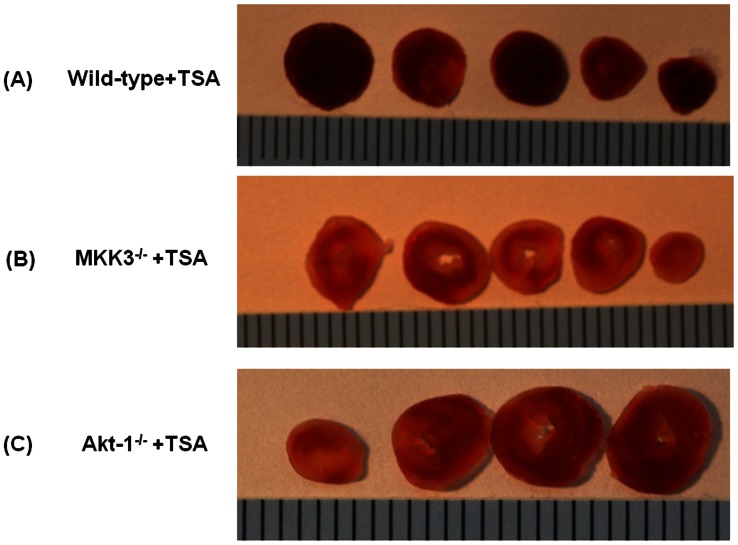
Myocardial infarct sizes. Representative sections of a heart demonstrating post-ischemic infarct size 30 min after treatment with TSA in wild-type(A), MKK3^−/−^ (B) and Akt-1^−/−^ (C) mice exposed to myocardial ischemia and reperfusion injury. At the end of the experimental protocol as described in Methods, the hearts were sliced into 4–5 sections and stained with 2,3,5-triphenyltetrazolium chloride followed by fixation in formalin. Viable areas are stained brick red, whereas infarcted areas are gray or white.

Myocardial infarct size, an index of irreversible myocardial injury, was assessed. Following TSA treatment, the infarct size in the wild-type was significantly decreased as compared to wild type vehicle-treated group. However, disruption of MKK3 mitigated the infarct-sparing effect elicited by trichostain A (p<0.05, **Figure 2** **and** **3**). In the absence of trichostatin A treatment, there was no difference in infarct size among MKK3^−/−^ and wild type vehicle-treated group. Likewise, the reduction of infarct size elicited by trichostain A was absent in Akt-1^−/−^ mice (p<0.05, **Figure 2** **–** **3**). There was no difference in infarct size between Akt-1^−/−^ and wild type vehicle treated group without trichostatin A treatment. The data suggest that the reduction of infarct size by HDAC inhibition requires MKK3 and Akt-1.

**Figure 3:**
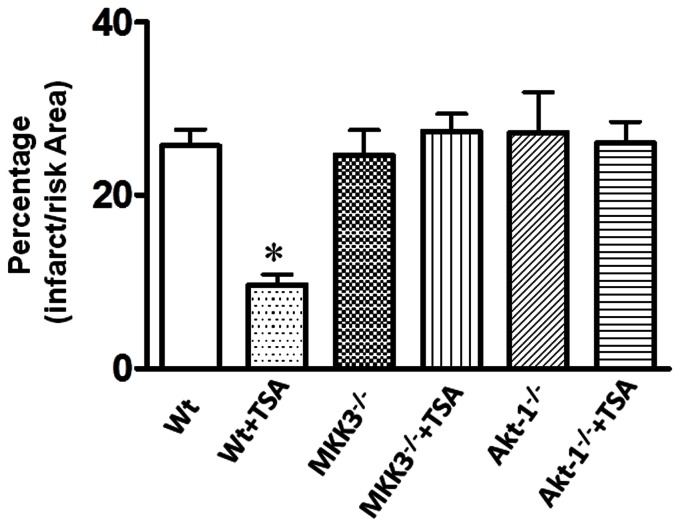
The effect of TSA on myocardial infarct size in wild-type, MKK3^−/−^ and Akt-1^−/−^ mice. Note that there is a significant greater viable area in the sections of the heart obtained from TSA-treated wild type mice as compared to wild type vehicle-treated group, TSA+ MKK3^−/−^ mice, TSA+Akt-1^−/−^ mice. Values represent mean ± SE. *p<0.05 *vs* wild-type vehicle-treated mice, TSA+ MKK3^−/−^ mice and TSA+Akt-1^−/−^ mice (n = 4–5 per group).

### Post-ischemic Ventricular Function

**Figure 4:**
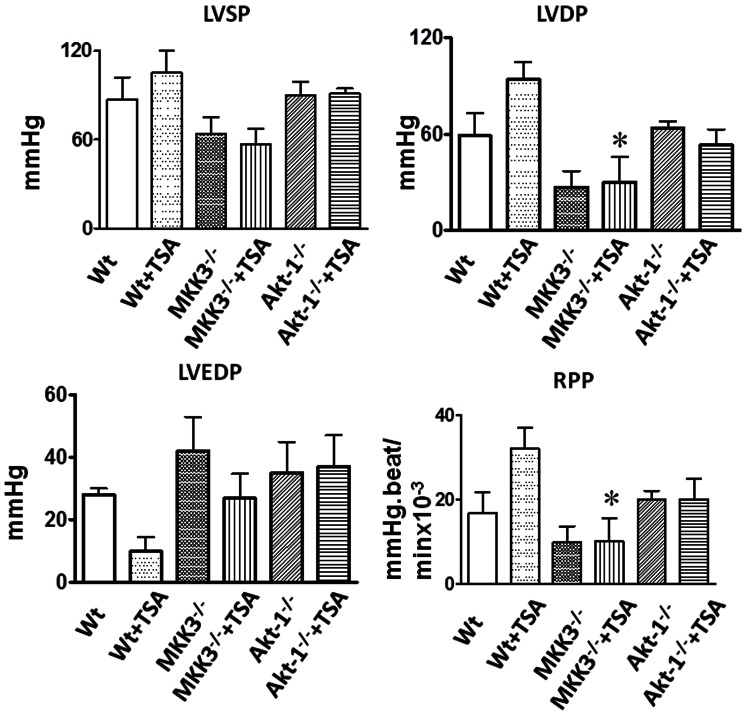
Post-ischemic ventricular functional recovery. Absence of cardiac functional recovery in MKK3^−/−^ and Akit-1^−/−^ mice treated with trichostatin A. Left ventricular (LV) function was assessed in isovolumetric hearts. The measured parameters include LV systolic pressure (LVSP), developed pressure (LVDP), and rate pressure product (RPP) where LVDP is systolic pressure minus LV end-diastolic pressure (LVEDP). Values represent mean ± SE (n = 4–5 per group), *p<0.05 *vs* Wt+TSA.

**Figure 5:**
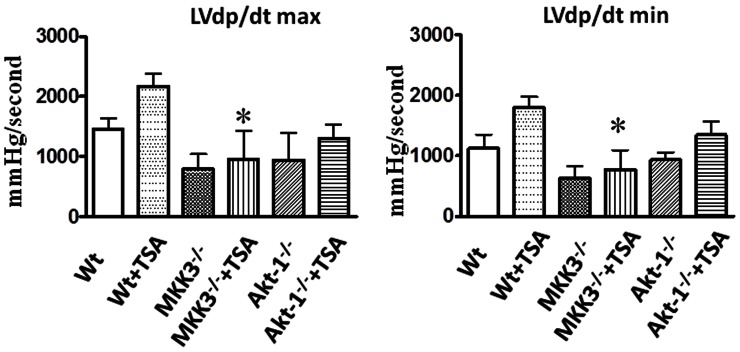
Effects of TSA on LV-dP/dt max and LV-dP/dt min. Values represent mean ± SE, *p< 0.05 *vs* Wt+TSA (n = 4–5 per group).

**Figure 6:**
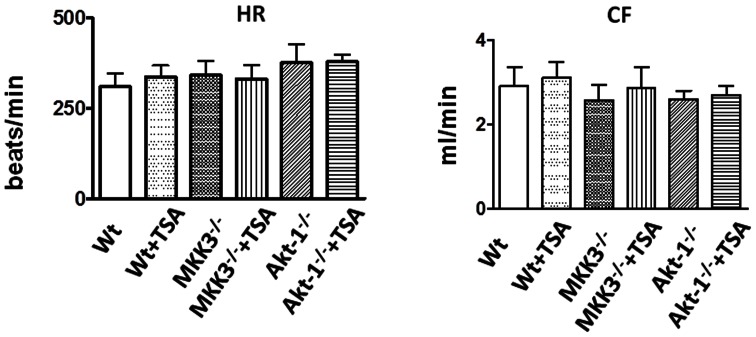
Effects of TSA on heart rates and coronary effluents in post-ischemic myocardium. CF: coronary effluent; HR: heart rate, values represent mean ± SE (n = 4–5 per group). There were no significance differences among groups.

In order to see whether MKK3 and Akt-1 are involved in HDAC inhibition-induced myocardial protection, MKK3 and Akt-1 deficient mice were treated with trichostatin A. As shown in **Figure 4**, following TSA treatments, the trend in LVDP, LVEDP, and rate pressure products (RPP) improved although these functional recoveries did not reach statistical differences as compared to vehicle-treated groups. The improvement in the LVDP in HDAC inhibition-treated mice in MKK3^−/−^ mice was significantly lower as compared to wild-type. Likewise, recovery of LVEDP elicited by HDAC inhibition in wild-type mice disappeared in Akt-1^−/−^ and MKK3^−/−^ mice that received TSA treatments. In addition, recovery of RPP in wild-type mice treated with TSA was mitigated in Akt-1^−/−^ mice and was significantly lower in MKK3^−/−^ mice treated with TSA. Post-ischemic recovery of LV dP/dt max and LV dP/dt min in wild-type mice was increased following TSA treatment (**Figure 5**). However, improvements in both LV dP/dt max and LV dP/dt min in wild-type mice following TSA treatments were not demonstrated in TSA-treated Akt-1^−/−^ mice, but these values were significantly lower in TSA-treated MKK3^−/−^ mice. Post-ischemic recovery of heart rate and coronary flow was similar in all the groups (**Figure 6**). In the absence of trichostatin A treatments, both MKK3^−/−^ mice and Akt-1^−/−^ mice did not show a difference in cardiac functional recovery as compared to wild-type vehicle-treated mice. These results indicate the necessity of MKK3 and Akt-1 in cardiac protection elicited by HDAC inhibition.

### MKK3 Acetylation in Myocardium

**Figure 7:**
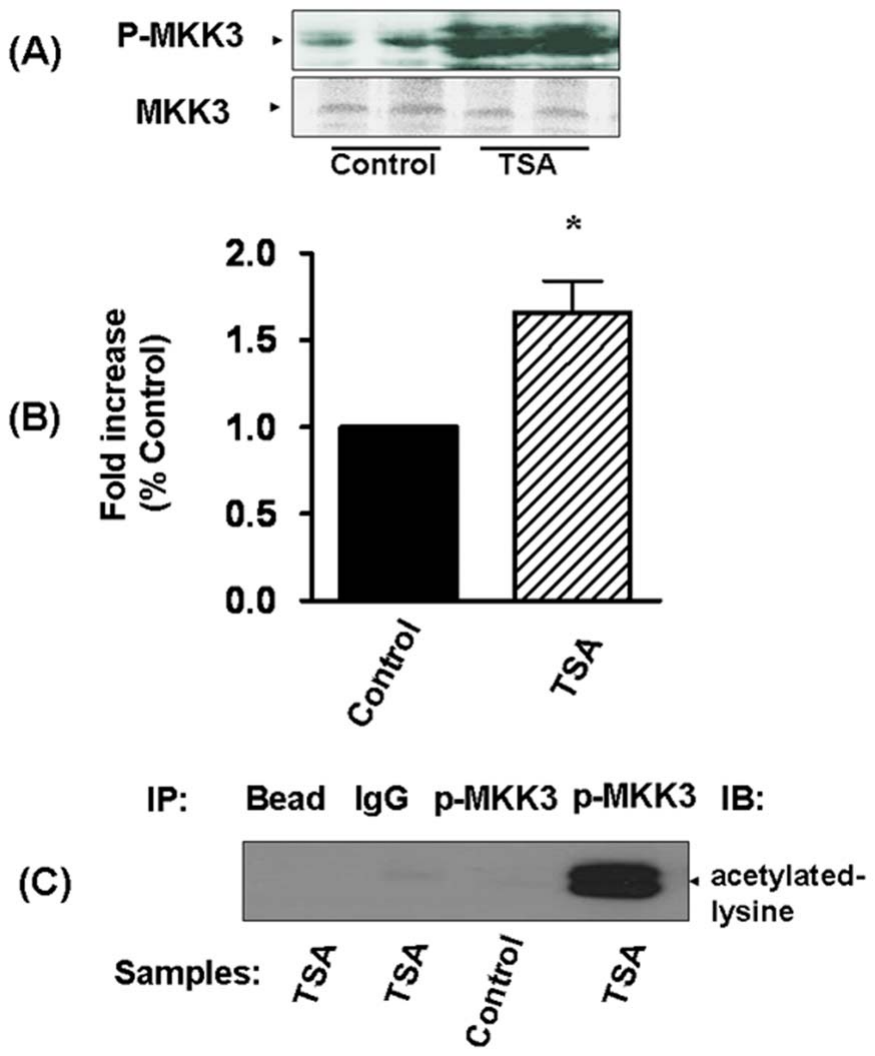
The effects of TSA on acetylation and phsophorylation of MKK3 in myocardium. The increased acetylated and phosphorylated MKK3 in myocardium after TSA treatment were observed. (A): TSA treatment increased MKK3 phosphorylation in myocardium; (B): The densitometric signal of phosphorylated MKK3 level, the densitometric signal was normalized to the control group and expressed as a percentage; (C): Immunoprecipitation and Western blot: myocardial tissues were collected at 30 min after TSA treatment. Results are mean ± SE (n = 3/per group), *p<0.05 *vs* control.

**Figure 8:**
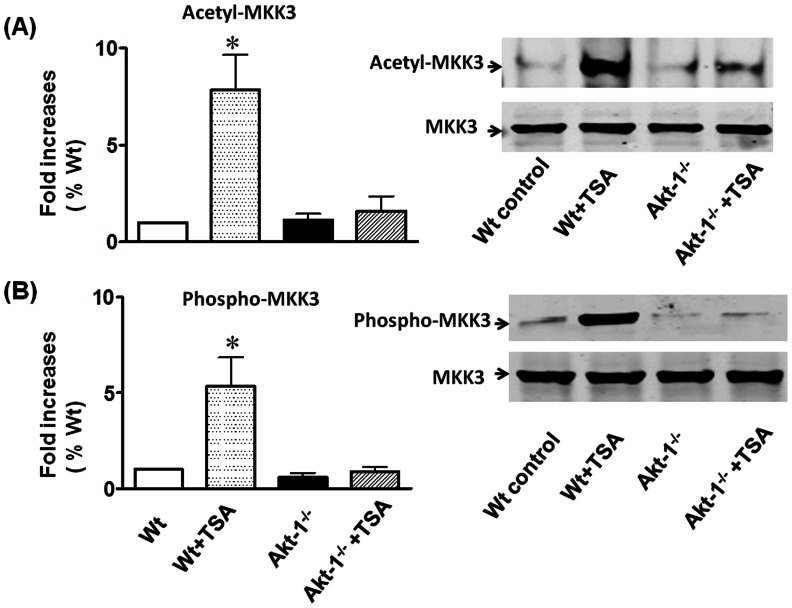
The disruption of Akt-1 abrogates TSA-induced acetylation and phosphorylation of MKK3 in the myocardium. The increased acetylated and phosphorylated MKK3 levels in myocardium after TSA treatment were observed in wild type, but not in Akt-1^−/−^ mice. The details of immunoprecipitation and Western blot are described in the Methods. (A): Disruption of Akt-1 abrogated TSA-induced MKK3 acetylation in myocardium (left, the densitometric signals; right, representative gel). (B): Disruption of Akt-1 abrogated TSA-induced MKK3 phosphorylation in myocardium (left, the densitometric signals; right, representative gel). The densitometric signal was normalized to the wild type control group and expressed as a percentage. Results are shown as mean ± SE (n = 3/per group), *p<0.05 *vs* Wt control, TSA+Akt-1^−/−.^

We examined myocardial MKK3 in hearts treated with TSA. As shown in **Figures 7A**, TSA treatment resulted in a transient MKK3 phosphorylation as compared to control group. The results of densitometry analysis of MKK3 phosphorylation increased significantly following TSA treatment, demonstrating that TSA treatment stimulates the phosphorylation of MKK3 (**Figure 7B**). It is well known that TSA generally results in a hyperacetylation of downstream target, we sought to examine whether TSA treatment induces the acetylation of MKK3. As shown in **Figure 7C**, as compared to control group, the immunoprecipitation assay showed that administration of TSA effectively caused the acetylation of MKK3. There was no MKK3 acetylation signal in IgG negative control reaction, suggesting that HDAC inhibition stimulates MKK3 acetylation signaling in association with myocardial protection. Furthermore, to examine whether the disruption of Akt-1 would abrogate acetylation as well as phosphorylation of MKK3, we examined the contents of acetylated and phosphosphorylated MKK3, respectively. As shown in **Figure 8** **,** in the absence of Akt-1, TSA treatments did not result in increases in acetylation and phosphorylation of MKK3, suggesting that MKK3 acetylation and phosphorylation require Akt-1.

**Figure 9:**
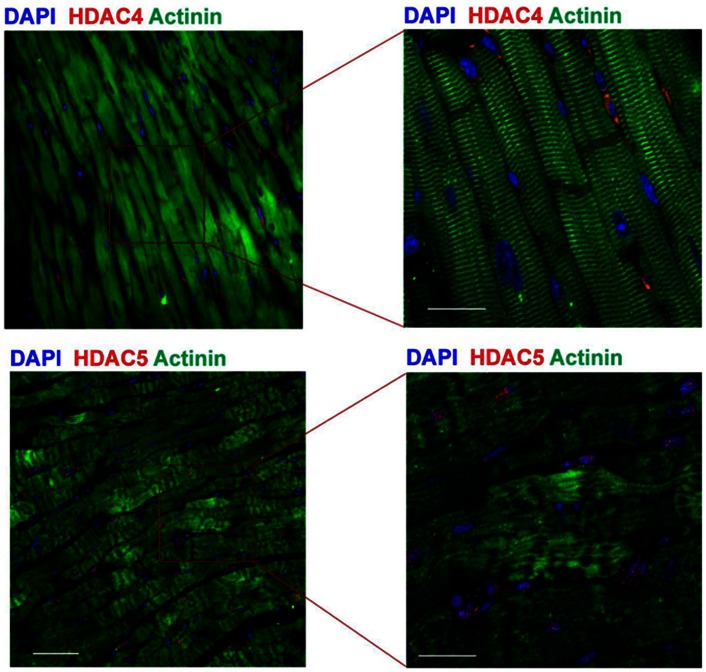
The presence of HDAC4 and HDAC5 in mouse myocardium. The myocytes were stained with anti-sarcomeric actinin (green), HDAC4 and HDAC5 proteins were stained with anti-HDAC4 and HDAC5 (red). Nuclei were stained with 4′6-diamidino-2-phenylindole (DAPI, blue). Images show an overlay of myocytes, HDAC4 and HDAC5 and nuclei, Bar = 50µm.

In addition, abundant amounts of cytosolic class II HDAC4 and HDAC5 were demonstrated in adult mouse heart (**Figure 9**), TSA-treated heart demonstrated a diminished level of HDAC4 and HDAC5 (data not shown), supporting that class II HDACs serves as a potentially important target in modulation of acute myocardial ischemia and reperfusion injury through HDAC inhibition.

## Discussion

### Salient Findings

Growing evidence from our recent studies indicates that HDAC inhibition serves as a novel signaling pathway to confer myocardial protection following ischemia and reperfusion. The mechanism that underlies the HDAC inhibition-induced protective effect remains unknown. We recently have found that HDAC inhibition preserved cardiac performance and prevented myocardial remodeling in infarcted myocardium, which is associated with the activation of p38 and Akt-1. It remains unclear whether MKK3 and Akt-1 are required for HDAC inhibition to induce a cardioprotective effect. In this investigation, using genetic animal models and pharmacological approaches we demonstrated that the deletion of MKK3 and Akt-1 abrogated myocardial protection of HDAC inhibition. Specifically, our study reveals that: 1) HDAC inhibition resulted in an improvement in ventricular functional recovery following myocardial injury, but HDAC inhibition-induced myocardial recovery was abrogated by elimination of MKK3 and Akt-1; 2) HDAC inhibition reduced myocardial infarct size, but infarct sparing effects of HDAC inhibition was abolished by disruption of MKK3 and Akt-1 as compared to the wild type mice; 3) The inhibition of HDAC resulted in increases in MKK3 acetylation and MKK3 phosphorylation, which is abrogated by disruption of Akt-1. Taken together, this study is the first demonstration to reveal that MKK3 and Akt-1 play an essential role in HDAC inhibition–induced cardioprotection.

HATs and HDAC govern gene expression patterns by being recruited to target genes through association with specific transcription factors [32]–[33]. We and others have previously demonstrated that inhibition of HDACs with a selective inhibitor confers cardioprotection and blocks cardiac hypertrophy [14], [15], [31]–[35]. Recently using a myocardial infarction model, our observation exhibited that HDAC inhibition manifested an anti-hypertrophic effect, prevented cardiac modeling and improved myocardial functional recovery, which was associated with the activation of Akt-1 signaling [35]. Consistent with findings in infarcted myocardium, in acute myocardial ischemia and reperfusion model, our observation also showed the HDAC inhibition-induced cardiac protection was lost in Akt-1^−/−^ mice. Additionally, we also found that HDAC inhibition effectively stimulation of Akt-1 phosphorylation (data not shown). These conclusions suggest that stimulation of Akt-1 may serve as a common pathway for HDAC inhibition to confer myocardial protection in both acute and post-MI hearts. Recent observation indicated that Akt-1 could be acetylated in lysine residues in response to stimulation of acetyltransferase [36]. It is well known that HDAC inhibitors resulted in hyperacetylation that participates in modulating the post-modification of downstream targets. It is unknown, but likely that Akt-1 acetylation could be stimulated following HDAC inhibition in the myocardium. Given that long term administration of HDAC inhibitor from our recent studies also resulted in an activation of Akt-1 phosphorylation [35], it will be interesting to elucidate whether acetylation and phosphorylation of Akt-1 play a distinct role between acute ischemic injury and post-infarcted myocardium.

The mitogen-activated protein kinases (MAPK) play a central role in the transmission of signals from cell surface receptors and environmental cues to the transcriptional machinery in the nucleus and are involved in cell growth, differentiation, and, transformation [37], [38]. The overexpression of MAP kinase kinase (MKK6), an upstream activator of p38, results in protection of cardiac myocytes from injury [39]. This was in agreement with the observations that an increase in p38 activation occurs in preconditioned hearts or reduces injury [40], [41]. We have demonstrated that the selective activation of p38 directly elicited a protective effect in the preconditioned hearts [26]. In addition, recent evidence has documented that pharmacologic activation of p38 induced myocardial protection against ischemic injury from preclinical large animal to human cardiac tissues [42]–[44], indicating the importance of pharmacologic activation of p38 pathway to achieve a therapeutic implication. However, it is unknown whether HDAC inhibition involved stimulation of MKK3 molecular signaling attributable to myocardial protection. Elimination of MKK3 completely abolished the ventricular functional improvement and reduction of myocardial infarct size following HDAC inhibition, establishing a cause and effect relationship between MKK3 and HDAC inhibition in the development of cardioprotection. Interestingly, our observation showed that HDAC inhibition significantly resulted in increase in MKK3 phosphorylation and acetylation, suggesting that MKK3 acetylation and phosphorylation is closely associated with HDAC inhibition-induced myocardial protection. However, how MKK3 acetylation mediates and/or correlates with the phosphorylation to regulate physiological function apparently merits future investigation. Moreover, the present study indicates that acetylation and phosphorylation of MKK3 following HDAC inhibition were blocked with elimination of Akt-1, suggesting that MKK3 acetylation and phosphorylation are dependent on Akt-1, and co-ordination between MKK3 and Akt-1 is essential in the development of cardioprotective effects against acute myocardial ischemia and reperfusion injury. Furthermore, it is also essential to implement in vivo myocardial I/R model to explore the implications of MKK3 and Akt-1 following HDAC inhibition in mediating functional performance as well as remodeling, which could provide further insight to understanding the mechanisms of I/R and translational evidence for clinical therapy.

In conclusion, our study is the first demonstration to reveal that MKK3 and Akt-1 involve acquisition of preconditioning effect elicited by HDAC inhibition. HDAC inhibition resulted in improvement in ventricular function and reduction of myocardial infarct, which was absent by the disruption of MKK3 and Akt-1. Furthermore, HDAC inhibition induced increase in acetylation and phosphorylation of MKK3. Identification of this novel signaling pathway not only provides new insight into the mechanism of pharmacologic preconditioning, but also holds great promise in developing a new therapeutic strategy for the ischemic heart.
