# Correction: HDAC Inhibition Elicits Myocardial Protective Effect through Modulation of MKK3/Akt-1

**DOI:** 10.1371/annotation/29be7977-2e22-40cd-8a71-b3fbb99de9d5

**Published:** 2013-07-01

**Authors:** Ting C. Zhao, Jianfeng Du, Shugang Zhuang, Paul Liu, Ling X. Zhang

The name of the third author was spelled incorrectly. The correct name is: Shougang Zhuang. 

